# Anatomical variation in bifurcation and trifurcations of sciatic nerve and its clinical implications: in selected university in Ethiopia

**DOI:** 10.1186/s13104-015-1626-6

**Published:** 2015-11-02

**Authors:** Birhane Alem Berihu, Yared Godefa Debeb

**Affiliations:** Department of Anatomy and Histology, Institute of Biomedical Sciences, College of Health Sciences, Mekelle University, Mekelle, Ethiopia; Department of Physiology, Institute of Bio–Medical Sciences, College of Health Sciences, Mekelle University, Mekelle, Ethiopia

**Keywords:** Sciatic nerve, Tibial and common peroneal nerves, Trifurcation of the sciatic nerve, Bifurcation of the sciatic nerve

## Abstract

**Background:**

The Sciatic nerve is the widest nerve of the body. It consists of two components, namely: the tibia and the common peroneal components derived from the ventral rami of L4 to S3 spinal nerves of the lumbosacral plexus. It exits the pelvis through the greater sciatic foramen below the Piriformis muscle and descends between the greater trochanter of the femur and ischial tuberosity of the pelvis to the knee. The purpose of this study is to identify the course and variations in branching pattern of the sciatic nerve which may lead to various clinical manifestations.

**Methods:**

Twenty-eight formalin fixed cadavers comprising of 56 lower limbs are used for this study, of which six specimens were female cadavers. Dissection of gluteal region and posterior compartment of the thigh was done to expose the sciatic nerve. Variations in the sciatic nerve anatomy; their relationship to Piriformis muscle and a point of bifurcation and trifurcation were noted and recorded.

**Results:**

Forty-two lower limbs (75 %) showed normal anatomy of sciatic nerve. Fourteen regions (25 %) showed variations in the sciatic nerve, of which six regions (11 %) showed a variation of the sciatic nerve in relation to Piriformis muscle, three regions (5 %) showed trifurcation of the sciatic nerve and five regions (9 %) showed variation in the origin of the sural nerve.

**Conclusion:**

The knowledge regarding the level of division and distribution of the sciatic nerve and its location is of great importance. The sciatic nerve is frequently involved in daily medical practice of neurology, orthopedics, rehabilitation and anesthesia. Its long course makes it vulnerable to nerve injury. Even in this era the cadaver is the best means to study anatomy. It emphasizes proper clinical implications, for the surgeons to practice efficient surgical recombination and avoid errors.

## Background

Sciatica is a Greek word derived from “Ischiadichus” and hence it is called as ischiadic nerve. The sciatic nerve is the widest nerve of the body, consisting of two components, namely: the tibia and the common peroneal component, both of which initially form a common trunk from the lumbosacral plexus. The tibial component is formed from the ventral branch of ventral rami of L4 to S3 spinal nerves. The common peroneal component is formed from the dorsal branches of ventral rami of L4 to S2 spinal nerves [1]. It exits the pelvis through the greater sciatic foramen below the Piriformis muscle and descends between the greater trochanter of the femur and ischial tuberosity of the pelvis, at the back of the thigh, divides into the tibial nerve and common peroneal (fibular) nerves at a varying level proximal to the knee. Superiorly it lies deep to the gluteus maximus muscle, resting first on the posterior ischial surface with the nerve to the quadratus femoris lying between them. It crosses posterior to the obturator internus muscle, the gemeli muscle and quadratus femoris muscle, separated by the latter from obturator externus muscle and the hip joint. Medially, it is accompanied by the posterior femoral cutaneous nerve and the inferior gluteal artery. Distally, it lies behind adductor magnus and is crossed posteriorly by the long head of biceps femoris. It corresponds to a line drawn from just medial to the midpoint between the ischial tuberosity and greater trochanter to the apex of the popliteal fossa.

Articular branches arise proximally to supply the hip joint through its posterior capsule; these are sometimes derived directly from the sacral plexus. Muscular branches are distributed to the biceps femoris muscle, semitendinosus muscle, semimembranosus muscle and the ischial part of adductor magnus muscle. The point of division of the sciatic nerve into the tibial nerve and the common peroneal nerve is very variable. The common site is at the junction of the middle and lower thirds of the thigh, near the apex of the popliteal fossa. The division may occur at any level above this, though rarely below it. It is not uncommon for the major components to leave the sacral plexus separately [1]. The sciatic nerve supplies the knee flexors and all the muscles below the knee, so that a complete palsy of this nerve results in a flail foot and severe difficulty in walking. This is rare and usually related to trauma. The nerve is vulnerable in posterior dislocation of the hip. As it leaves the pelvis, it may become entrapped called the Piriformis syndrome, a common anatomical variant but an extremely rare entrapment neuropathy).

External compression over the buttock (e.g. In patients who lie immobile on a hard surface for a considerable length of time) can injure the nerve. The most common cause of serious sciatic nerve injury (and consequent major medicolegal claims) is iatrogenic.

It may be injured from misplaced therapeutic injections into the gluteus maximus muscle. Sciatic nerve palsy also occurs after total hip replacement or similar surgery in 1 % of cases. This injury can be due to a sharp injury burning from bone cement, traction from instruments, and manipulation of the hip, inadvertent lengthening of the femur, or hematoma surrounding the nerve or within its soft tissue coverings. Early surgical exploration and evacuation of hematomas can reverse the nerve lesion. Complete sciatic nerve palsy is very rare. For some anatomical reason, the common peroneal nerve is more usually nerve affected, relating to a foot drop and a high stepping gait [1]. Several authors have reported variations on its division into the tibial and common peroneal nerve from the sacral plexus to the lower part of the popliteal space [1, 4, 6–10, 15–21]. Also, there are case reports on unusual trifurcation of the sciatic nerve on the back of the thighs [11, 13, 27, 28]. These anatomical variations may contribute to Piriformis syndrome, sciatica and other nerve problems [11]. This should be taken into account by clinicians who are planning interventions around the sciatic nerve and its division in the lower extremity. Hence, this study aims at observing the course and variations in the branching pattern of the sciatic nerve.

## Methods

Fifty-six lower limbs (44 males and 12 females) from 28 cadavers (22 male and 6 female) formalin fixed cadavers without any gross pathology were used for this study. The cadavers belong to the Department of Anatomy in Ethiopian Governmental Medical Colleges (Mekelle University, Adigrat University, and Axum University). Appropriate consent was obtained from Mekelle University College of Health Sciences; Health Research Ethics Review Committee (HRERC) for the use of the cadavers in clinical research and to the other universities where the data were collected. This study was done during the routine dissection classes for medical undergraduate students over a period of 1 year and 6 months from 2014 to 2015. All the cadavers were numbered in a sequential manner. Dissection of gluteal region was done by exposing and cleaning of the gluteus maximus muscle to show the structures covered by it. Piriformis muscle and its the relation to the sciatic nerve and its branches were observed and recorded. Dissection of the posterior compartment of the thigh was also done to observe the course of the sciatic nerve, its branching variations and a point of bifurcation and trifurcation were observed and recorded.

## Study design and statistical analysis

In the present study, we use non probability sampling (convenience sampling) method, in which the units during the time of data collection were selected. All available cadavers without gross pathology were included in the paper and all the data collected were recorded. The simple statistical method was also used for analysis for possible variations. This analysis identified sciatic nerve variations with high divisions and lower divisions in detecting the presence of bifurcation and trifurcation of the sciatic nerve. The frequency of anatomical variations was analyzed in an independent prospective sample collected from the gluteal region and the posterior thigh region of the lower limbs. Anatomical variations of sciatic nerve were documented in percentage. Findings are presented in frequency tables and figures.

## Results

Twenty-eight formalin fixed cadavers comprising of 56 lower limbs were used for this study. Among them 42 lower limbs (75 %) showed a normal anatomy of the sciatic nerve. Fourteen lower limbs (25 %) showed variations in the sciatic nerve. Of the fourteen lower limbs (25 %), the six lower limbs (11 %) show variations of the sciatic nerve in relation to piriformis muscle, of which five lower limbs (9 %), Common peroneal component and tibial component arises separated below the Piriformis and rejoin posterior to quadratus femoris muscle and bifurcate at the superior angle of popliteal fossa. The rest one lower limb (2 %), the common peroneal component emerges above the Piriformis and tibial component emerges below the Piriformis and descends separately along their course.

**Fig. 1:**
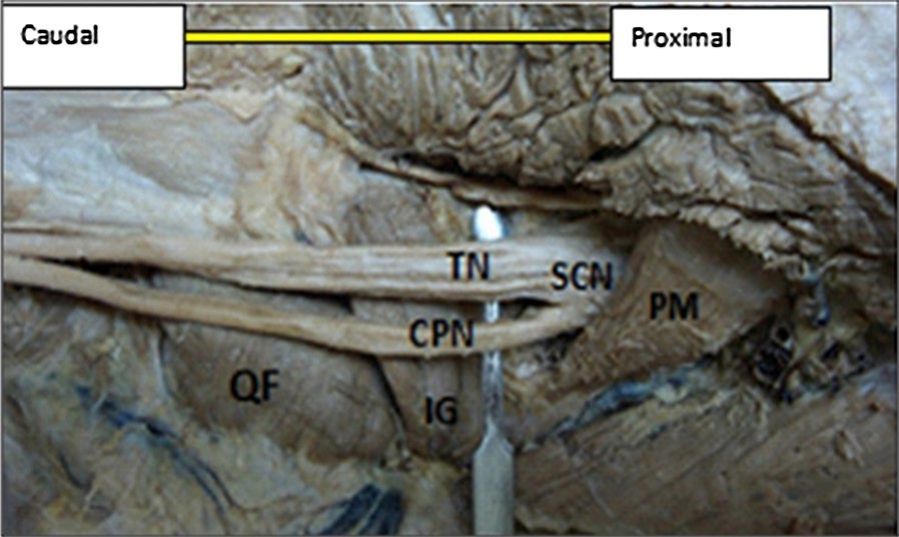
Photograph of the dissected male cadaver of the right gluteal region and *back* of the thigh showing high bifurcation of the right sciatic nerve (SN) by 1 cm below the *lower border* of Piriformis muscle (PM). The nerve is divided into the tibial nerve (TN) and common peroneal nerve (CPN). *QF* quadratus femoris muscle, *IG* inferior gemellus muscle

**Fig. 2:**
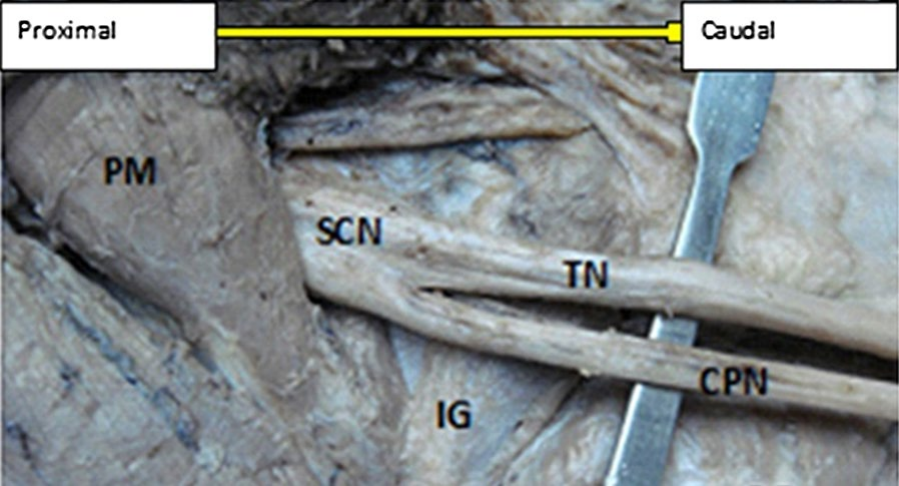
Photograph of the dissected female cadaver of the left gluteal region and *back* of the thigh showing high bifurcation of the left sciatic nerve (SN) by 2 cm below the lower border of Piriformis muscle (PM). The nerve is divided into the tibial nerve (TN) and comm on peroneal nerve (CPN). *QF* quadratus femoris muscle, *SG* superior gemellus muscle, *IG* inferior gemellus muscle

**Fig. 3:**
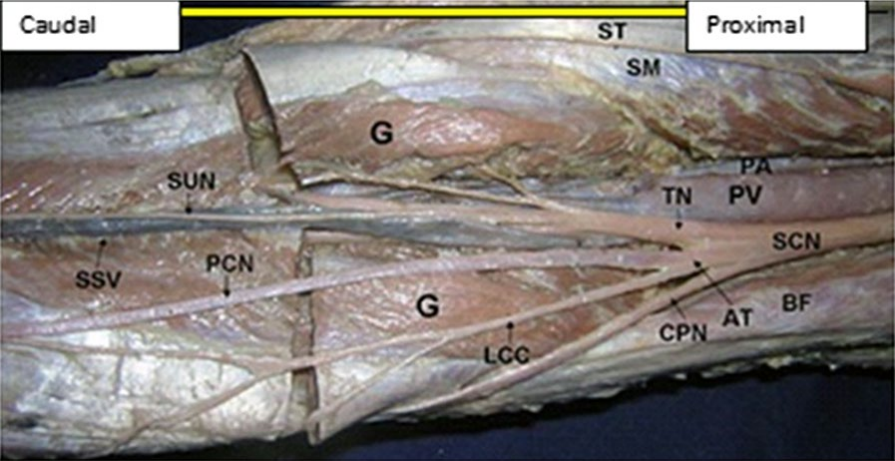
Dissection of the right popliteal fossa showing trifurcation of the sciatic nerve. *SCN* sciatic nerve dividing low in the popliteal fossa, *TN* tibial nerve, *CPN* common peroneal nerve, *AT* abnormal trunk, *LCC* lateral cutaneous nerve of the calf, *PCN* peroneal communicating nerve, *SUN* sural nerve, *PV* popliteal vein, *SSV* small saphenous vein, *PA* popliteal artery, *G* two heads of gastrocnemius muscle, *ST* semitendinosus, *SM* semimembranosus, *BF* biceps femoris

**Fig. 4:**
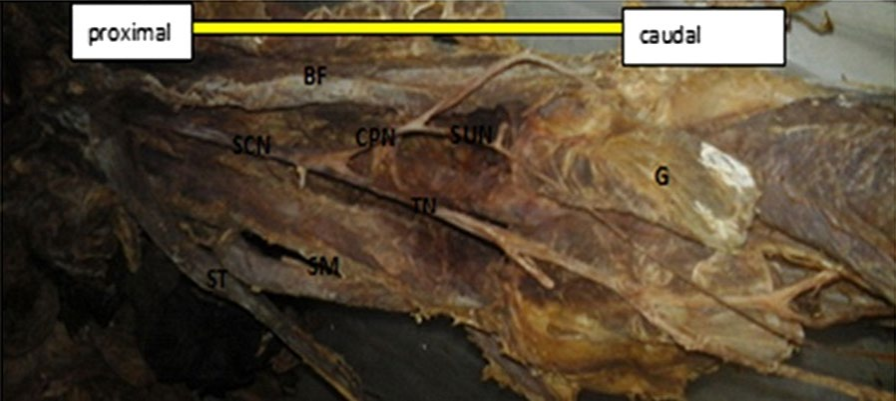
Dissection of the left popliteal fossa, showing furcation of the sciatic nerve. *SCN* sciatic nerve dividing low in the popliteal fossa, *TN* tibial nerve, *CPN* common peroneal nerve, *SUN* sural nerve, *PVV* popliteal vessel, *G* two heads of gastrocnemius muscle, *SM* semimembranosus, *BF* Biceps Femoris, *ST* S emitendinous

**Fig. 5:**
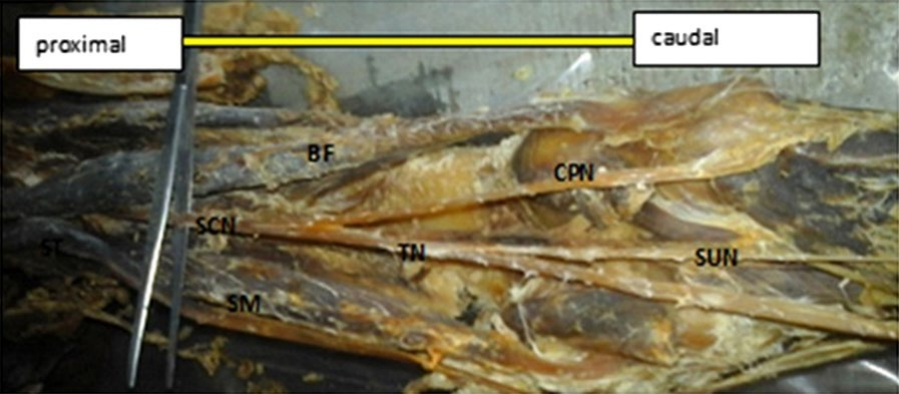
Dissection of the left popliteal fossa showing division of the sciatic nerve. *SCN* sciatic nerve dividing low in the popliteal fossa, *TN* tibial nerve, *SUN* sural nerve, *CPN* common peroneal nerve, *PVV* popliteal vessel, *SM* semimembranosus, *BF* Biceps Femoris, *ST* Semitendinous

**Fig. 6:**
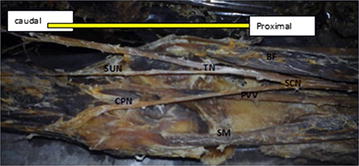
Dissection of the left popliteal fossa showing division of the sciatic nerve. *SCN* sciatic nerve dividing low in the popliteal fossa, *TN* tibial nerve, *SUN* sural nerve, *CPN* common peroneal nerve, *PVV* popliteal vessel, *SM* semimembranosus, *BF* Biceps Femoris, *ST* Semitendinous

**Fig. 7:**
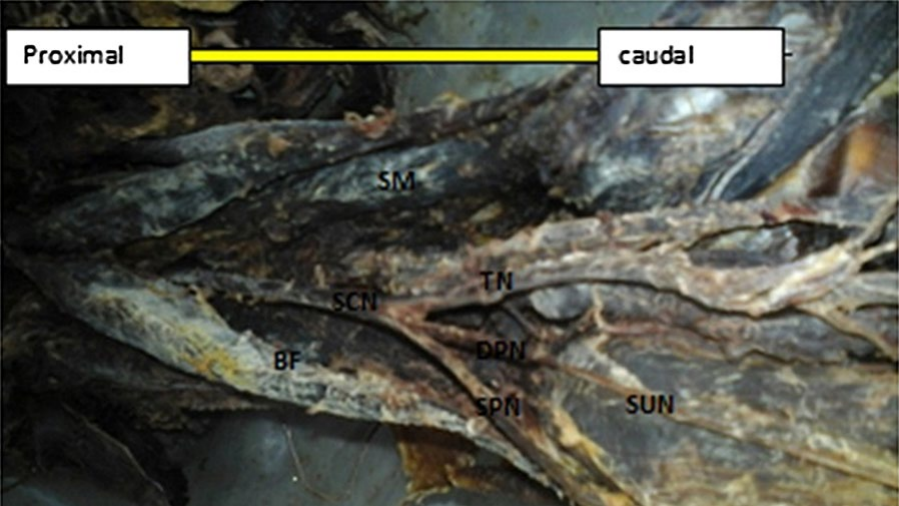
Dissection of the female left popliteal fossa showing trifurcation of the sciatic nerve. *SCN* sciatic nerve dividing low in the popliteal fossa, *TN* tibial nerve *DPN* deep peroneal nerve, *SPN* superficial peroneal nerve, *SM* semimembranosus, *BF* Biceps Femoris, *SUN* sural nerve

Moreover, the rest eight lower limbs (14 %), shows variations of the sciatic nerve in relation to popliteal region of the thigh. Of these eight lower limbs (14 %), the three lower limbs (5 %) showed trifurcation of sciatic nerve into three major divisions (tibial nerve, common peroneal nerve and an unusual trunk) in the middle of the popliteal fossa on the right side of the male lower limb. The unusual trunks divided into the lateral cutaneous nerve of the calf and peroneal communicating nerve. Additionally, one female lower limb showed trifurcation of sciatic nerve into tibial, superficial and deep peroneal nerves on the left side at the superior angle of the popliteal fossa. Furthermore, of these eight lower limbs (14 %), the five lower limbs (9 %) showed three branches: tibial, common peroneal and sural nerves. In this case the origin of the sural nerve on three lower limbs originate directly from common peroneal nerve on the left side and on the other two lower limbs is originated directly from the tibial nerve on the left and right side. Table 1 depicting the percentage in level of division of the sciatic nerve in the present study. Figures 1, 2, 3, 4, 5, 6, 7 illustrates the sciatic nerve variations observed in this study.

**Table 1:** Depicting the percentage in level of division of the sciatic nerve in the present study

Item	Level of division of SCN					
Sample specimens	Normal course and distribution N (%)	Bifurcation at gluteal region N (%)	Trifurcation in popliteal region on left side N (%)	Trifurcation in popliteal region on on right side N (%)	Formation of SUN only form TN and only from CPN N (%)	Total no of specimen N (%)
Male specimen	32 (57)	5 (9)	0	2 (3.6)	5 (9)	44 (79)
Female specimen	10 (17.9)	1	1	0	0	12 (21)
Total	42 (75)	6 (10.7)	1	2 (3.6)	5 (9)	56 (100)

## Discussion

The sciatic nerve, the largest branch of lumbosacral plexus is composed of ventral and dorsal divisions of ventral rami of L4 to S3 spinal nerves. The sciatic nerve is formed when the large dorsal component of the sacral plexus (common fibular nerve) and the ventral component (tibial nerve) move downward close together [1, 6, and 7] and hence the common peroneal and tibial components can separate from each other at various levels from their origin [1, 6, and 7]. Various studies are available in the literatures regarding sciatic nerve variations. The present study shows five lower limbs (9 %) with two terminal branches of sciatic nerve emerging below the Piriformis muscle directly and descends separately throughout their course and in one lower limb (2 %) the common peroneal nerve emerges above the Piriformis muscle and the tibial nerve emerges below Piriformis. In a study by Shewale et al., 2 % of the specimens showed the common peroneal and the tibial nerve emerge separately below the Piriformis muscle. The tibial nerve was in the rootlet stage [17]. 15–30 % of the sciatic nerve variations in relation to Piriformis muscle are reported in the previous studies [25]. Beaton and Anson have classified the relationship of sciatic nerve to the Piriformis muscle in 120 specimens in 1937 and 240 specimens in 1948 into six types [14, 26]. Their classification is as follows:

- Type 1: Undivided nerve below undivided muscle.
- Type 2: Divisions of nerve between and below undivided muscle.
- Type 3: Divisions above and below undivided muscle.
- Type 4: Undivided nerve between heads.
- Type 5: Divisions between and above heads.
- Type 6: Undivided nerve above undivided muscle.

In our study, according to Beaton and Anson’s classification of the relation of sciatic nerve variation to Piriformis muscle, 75 % (42 specimens) belongs to type I, 9 % (5 specimens) belongs to type II and 2 % (1 specimens) belongs to type III category. Specimens of type 4, type 5 and type 6 category of Beaton and Anson’s classification are not found in our study. A rare variation of common peroneal nerve passing under the Piriformis and tibial nerve passing under the superior gemellus has been reported by Babinski [5]. This variable is not found in the present study. Various studies have been reported about the high division of the sciatic nerve in the gluteal region. Shewale et al. [17], has reported 11 % of sciatic nerve division in the gluteal region. In study by Anbumani et al. [29], nine regions (18 %) of fifty lower limbs showed variations in the sciatic nerve, of which five regions (10 %) showed a variation of the sciatic nerve in relation to Piriformis muscle. Such cases were also reported by Shashtrakar et al. [30] in their study, where the existence of high SN division in 48 % of the cases. In some gluteal regions, the TN and CPN passed through infrapiriformis portion of the greater sciatic foramen with different sheaths (20 %) and other gluteal regions with high division shows the exit of TN and CPN through different routes. Similarly, Prakash et al. [22], has also reported 16.3 % sciatic nerve division in the gluteal region. Guvencer et al. [19], has also reported that 48 % of sciatic nerve divides in the gluteal region. Moore et al. [23], has been reported that common peroneal nerve passing through the Piriformis and the tibial nerve passing below Piriformis is at 12 % of specimens. Similarly, Chiba [24], has reported that common peroneal nerve passing through the Piriformis is in 34 % of cases in 514 extremities. These above mentioned findings are in line with our study which shows high division of the sciatic nerve found in 11 % of specimens. Previous anatomical studies have demonstrated 15 % variation in the relationship between the Piriformis and sciatic nerve [25].

Trifurcation of the sciatic nerve is rarely cited in the literatures. The sciatic nerve trifurcation were revealed in three lower limbs (5 %) in this present study. In this case, the sciatic nerve terminated in the middle of the popliteal fossa by giving three branches on the right side of the male lower limb (tibial nerve, common peroneal nerve and an unusual trunk). The unusual trunk divides into the lateral cutaneous nerve of the calf and peroneal communicating nerve and in one female lower limb, trifurcation of sciatic nerve into tibial, superficial and deep peroneal nerves were observed on the left side at the superior angle of popliteal fossa. This finding is supported by Nyak [2], who showed trifurcation of sciatic nerve into tibial, common peroneal and abnormal trunk in the middle of popliteal fossa and in a case report by Sharadkumar Pralhad Sawant [11] who reported bilateral trifurcation of the sciatic nerve in the middle of the popliteal fossa into tibial, superficial peroneal and deep peroneal nerves. Moreover, our study showed five lower limbs (9 %) had three branches: tibial, common peroneal and sural nerves. In this case, the sural nerve on three lower limbs originated directly from common peroneal nerve on the left side and the other two lower limbs originated directly from the tibial nerve on the left and right side. Our finding is similar to that of Tanvi et al. [12], who reported that the variant formation of sural nerve was found in the left leg of the 50-year-old male cadaver. In this case, the medial sural cutaneous nerve and the lateral sural cutaneous nerve, after respectively deriving from the tibial and common fibular nerve, were noticed to continue their course without any formation of a unique nerve trunk on the posterior side of left leg.

In this present study,

- The Sciatic nerve variations are found in 25 % of lower.
- 11 % of sciatic nerve variations are related to Piriformis.
- 75 % of type 1, 11 % of type 2 and 2 % of type 3 variations of the sciatic nerve in relation to Piriformis muscle are observed according to Beaton and Anson’s classification.
- The higher divisions of the sciatic nerve in the gluteal region immediately below the Piriformis muscle are in five lower limbs.
- Common peroneal component emerges above the Piriformis muscle and tibial component arises below the Piriformis muscle in one lower limb.
- Trifurcation of sciatic nerve into tibial nerve, common peroneal nerve and an unusual trunk in two lower limbs.
- Trifurcation of sciatic nerve into tibial, superficial and deep peroneal nerves in one lower limb.
- Formation of Sural nerve only from Tibial nerve in two lower limbs.
- Formation of Sural nerve only from common peroneal nerve in three lower limbs.

### Clinical significance

The variation of sciatic nerve division at different level of the body is challenging for diagnostic and therapeutic procedure in many clinical and surgical cases. Quick recognition of sciatic nerve variation makes surgical approaches more precise and effective. The anatomical variations in the level of division of the sciatic nerve require knowledge in nerve grafting for the most common surgical procedure in the popliteal region. It follows that describing the usual and the variant anatomical relationship of the sciatic nerve in the popliteal region. Its distribution is associated with different structures and blood vessels which are important to conduct a safe operation and good outcome [3, 5].

Data on the prospective variation in the anatomy of the sciatic nerve and sural nerve help surgeons in avoiding unnecessary complications. Knowledge of the unusual variety of sciatic nerve an in the present case enables the surgeon to find and preserve the nerve during fasciotomy, neurolysis, neuroma resection, or bony and soft tissue reconstruction. Surgical, Diagnosis (biopsy and nerve conduction velocity studies) and therapeutic interventions (nerve grafting) can be problematic due to the confuse on the origin of the may confuse the origin of the nerves and associated structures. Describing the origin, distribution and variation of the sciatic nerve in the lower extremities is potentially helpful for surgeons, radiologists and anatomists. Knowing the usual bifurcation anatomy as well as the possible variations of the sciatic nerve and its branches helps the radiologist and the surgeons to interpret correctly what they see and encounter in the work-up and treatment of patients with sciatica and other nerve problems. This knowledge is likely to result in more accurate, expeditious and effective diagnosis and treatment of diseases related to nerve problems with consequent reduction in paralysis of the extremities and disabilities. Therefore, the accomplishment of this study contributes to the subject of sciatic nerve variation, by confirming the previous studies and emphasizing the need of a profound anatomical knowledge and good clinical outcomes.

## Conclusion

The knowledge regarding the level of division and distribution of the sciatic nerve and its location is of great importance. The sciatic nerve is frequently involved in daily medical practice of neurology, orthopedics, rehabilitation and anesthesia. Its long course makes it vulnerable to nerve injury. Even in this era the cadaver is the best means to study anatomy. The main essence of this study is to get some information on the variation of the sciatic nerve anatomy. The principal author was confronted with sciatic nerve and its bifurcation and trifurcation variations in the routine cadaveric dissections while teaching the undergraduate students. This study systematically reviews previous studies from the literatures. It emphasizes proper clinical implications, for the surgeons to practice efficient surgical recombination and avoid errors. Treatment aimed at maximizing the mobility of the lower extremity.

## Abbreviations

SCN: sciatic nerve
TN: tibial nerve
CPN: common peroneal nerve
AT: abnormal trunk
LCC: lateral cutaneous nerve of the calf
PCN: peroneal communicating nerve
SUN: sural nerve
PV: popliteal vein
SSV: small saphenous vein
PA: popliteal artery
G: gastrocnemius muscle
ST: semitendinosus
SM: semimembranosus
BF: biceps femoris
